# Analysis of Coaxial Soil Cell in Reflection and Transmission

**DOI:** 10.3390/s110302592

**Published:** 2011-03-01

**Authors:** Mathew G. Pelletier, Joseph A. Viera, Robert C. Schwartz, Steven R. Evett, Robert J. Lascano, Robert L. McMichael

**Affiliations:** 1 Cotton Production and Processing Unit, USDA-ARS, Lubbock, TX 79403, USA; 2 Sensors Group Microsemi Corporation Lowell, MA 01851, USA; E-Mail: jviera@microsemi.com; 3 Soil and Water Management Research Unit, USDA-ARS, Bushland, TX 79012, USA; E-Mails: Robert.schwartz@ars.usda.gov (R.C.S.); Steve.evett@ars.usda.gov (S.R.E.); 4 Wind Erosion and Water Conservation Unit, USDA-ARS, Lubbock, TX 79403, USA; E-Mail: Robert.lascano@ars.usda.gov; 5 Plant Stress and Germplasm Development Unit, USDA-ARS, Lubbock, TX 79403, USA; E-Mail: Bobbie.mcmichael@ars.usda.gov

**Keywords:** TDR, cotton moisture, moisture sensing, permittivity, microwave sensing, microwave moisture

## Abstract

Accurate measurement of moisture content is a prime requirement in hydrological, geophysical and biogeochemical research as well as for material characterization and process control. Within these areas, accurate measurements of the surface area and bound water content is becoming increasingly important for providing answers to many fundamental questions ranging from characterization of cotton fiber maturity, to accurate characterization of soil water content in soil water conservation research to bio-plant water utilization to chemical reactions and diffusions of ionic species across membranes in cells as well as in the dense suspensions that occur in surface films. In these bound water materials, the errors in the traditional time-domain-reflectometer, “TDR”, exceed the range of the full span of the material’s permittivity that is being measured. Thus, there is a critical need to re-examine the TDR system and identify where the errors are to direct future research. One promising technique to address the increasing demands for higher accuracy water content measurements is utilization of electrical permittivity characterization of materials. This technique has enjoyed a strong following in the soil-science and geological community through measurements of apparent permittivity via time-domain-reflectometery as well in many process control applications. Recent research however, is indicating a need to increase the accuracy beyond that available from traditional TDR. The most logical pathway then becomes a transition from TDR based measurements to network analyzer measurements of absolute permittivity that will remove the adverse effects that high surface area soils and conductivity impart onto the measurements of apparent permittivity in traditional TDR applications. This research examines the theoretical basis behind the coaxial probe, from which the modern TDR probe originated from, to provide a basis on which to perform absolute permittivity measurements. The research reveals currently utilized formulations in accepted techniques for permittivity measurements which violate the underlying assumptions inherent in the basic models due to the TDR acting as an antenna by radiating energy off the end of the probe, rather than returning it back to the source as is the current assumption. To remove the effects of radiation from the experimental results obtain herein, this research utilized custom designed coaxial probes of various diameters and probe lengths by which to test the coaxial cell measurement technique for accuracy in determination of absolute permittivity. In doing so, the research reveals that the basic models available in the literature all omitted a key correction factor that is hypothesized by this research as being most likely due to fringe capacitance. To test this theory, a Poisson model of a coaxial cell was formulated to calculate the effective extra length provided by the fringe capacitance which is then used to correct the experimental results such that experimental measurements utilizing differing coaxial cell diameters and probe lengths, upon correction with the Poisson model derived correction factor, all produce the same results thereby lending support for the use of an augmented measurement technique, described herein, for measurement of absolute permittivity, as opposed to the traditional TDR measurement of apparent permittivity.

## 1. Introduction

Frequency domain analysis of soils, cotton lint, biological cells and media is rapidly gaining appreciation due to the ability to provide a true measurement of permittivity as opposed to an apparent permittivity that TDR analysis in the time domain provides. One of the driving factors behind this new trend is due to the recognition that in saline and high clay content soils, that the conductive soils dielectric loss has a profound impact on the measured apparent permittivity which causes large errors especially when temperature effects are taken into consideration.

Recent research [1], reports the use of frequency domain analysis for extending the use of TDR waveforms in conductive soils as an alternative solution to soils in which the standard TDR waveform return is lost due to excessive conductivity, which renders the traditional TDR technique unusable or highly inaccurate. In this report, they report the need for use of a correction equation to relate the measured scattering S11 parameters to the soil dielectric properties, was suggested by Clarkson [2]. Other researchers have also reporting success in the use of the Clarkson [2] correction Equations [3–5]. Of note was a cautionary report by Hoekstra and Delaney [6], of possible additional TE and TM propagation modes, in addition to the primary TEM mode, that would cause both phase and magnitude errors in the higher frequencies. Of further note is that both the Clarkson [2] formulation and the equivalent formulation by Kraft and Campbell [7,8] assumes TEM is the only mode of propagation in the coaxial or TDR cell. Additional similar research was reported [4], which hypothesized that additional propagation modes were a likely cause of perceived errors in their higher frequency measurements from their expected theoretical responses.

Also of interest is the work by Kraft [7], which utilized an impedance calculation of a transmission line terminated with an open ended coaxial soil-filled cell, which was derived along an alternative formulation linking measured reflection spectral response to the permittivity parameters, thereby providing a separate path to the correction of the measured spectrum to that of a free space plane wave propagation. This formulation has become popular of late and has been used with slight modifications by several researchers [8–11], and is typically referenced in the recent literature as the Campbell equation.

In comparing the two approaches taken by Clarkson and Kraft [2,7], and equivalently Campbell and researchers referencing him, of note is that they both used as their basis a transmission line terminated in a simple coaxial soil-filled cell. Of critical importance however is that neither of these researchers mentioned formulations to provide a means of correction for the other system components, *i.e.*, cable, cable length, connectors, multiplexors and instrument effects such as instrument to cable impedance miss-match, non-ideal pulse, time varying pulse, *etc*. One example of the fundamental need for such corrections are provided in reports of the effects of exterior equipment such as variations in coaxial cable lengths, transient suppressors, *etc*. [4,12,13], on the obtained measurements. Further evidence is provided by Jones and Or [1] and Freil and Or [5], by their encouragement to utilize permittivity standards by which to judge obtained measurements against known standards. In moving toward utilization of permittivity standards, of critical need are calibration methods that couple models such as Clarkson [2], Kraft [7], and equivalently Campbell [8], to high quality calibration methods such as are utilized in the microwave engineering field for use in Network Analyzer measurements [7,16,17]. In moving forward towards resolving these issues, this research re-examines the open-terminated coaxial cell reflection from a theoretical basis to provide a sound background by which to re-examine the underlying assumptions of the models. This research then applies the developed theory towards confirmation via experimentation. Finally the theory is extended to provide a new model for use in through transmission measurements that are also inherently subjected to similar errors due to the impedance miss-match at the soil-cell to coax transition/s.

Specifically, this paper will demonstrate the impact of multi-reflection impedance miss-match on:

- measured permittivity in the frequency domain as compared to plane wave propagation in free space,
- impact on the waveform in the time domain,
- assumptions behind the Clarkson Equation,
- demonstrate the Campbell Equation provides numerically equivalent answers to the Clarkson [2] Equation.

Objectives: Derive a technique for the absolute measurement of permittivity from coaxial cells and,

- show the impact of miss-match impedance on waveforms,
- show derivation of the Clarkson [2] Equations,
- show where the main assumption, *i.e.*, pure reflection off the end of the probe is invalid by providing experimental evidence that the frequency at which point antenna radiation begins is occurring within the working bandwidth of the TDR system,
- show experimental results that don’t coincide with the predicted Clarkson [2] Equation,
- present a hypothesis, along with experimental results in support, to explain the response deviation from the Clarkson and equivalent Kraft and Campbell [2,7,8] Equations.

**Theory:** As the research community moves towards higher accuracy demands on TDR measurements, the natural evolution of the science will be to transition toward Network Analyzer measurements in the frequency domain due to the significant improvement in the accuracy and dynamic range the Network Analyzer provides over the traditional time domain TDR measurements, as well as the ability to utilize absolute permittivity standards which then enhances the accuracy and transferability of data between researchers. In moving from a measurement of apparent permittivity in the time domain towards a measurement of true permittivity and loss in the frequency domain, of critical importance is the removal of the response of the TDR or coaxial probe from the measurement. The following section details the electrodynamics to form a frequency response characterization for later removal of confounding affects to obtain a measurement of the true permittivity of the soil.

*Formulation of frequency response of through-transmission coaxial probe*. As the TDR probe is closely aligned to the coaxial cable, the analysis starts with the formulation for a coaxial cable by which to find the frequency response of the structure.

We note that for propagation of a free-space plane wave, in a source-less region that is directed only in the z direction, the form of the wave propagation can be shown to have the form of Equation 1, with the propagation coefficient γ as shown in Equation (2) [19], which can be derived from the phasor form of Maxwell’s electromagnetic equations (1):

(1) $$\begin{array}{c} \nabla \times H=j\varpi D+J\\ \nabla \times E=-j\varpi \mu H\\ \nabla \times H=j\varpi D+J=j\varpi ɛE+\sigma E=E\left(j\varpi ɛ+\sigma \right)=E\left(j\varpi \left(ɛ\prime -jɛ\prime \prime \right)+\sigma \right) \end{array}$$

where:

ɛ = ɛ′ − jɛ″: [complex permittivity (F/m)]
ɛ′ = ɛ_r_′ɛ_0_: [real component of complex permittivity]
ɛ″ = ɛ_r_″ɛ_0_: [imaginary component of complex permittivity]
D = ɛE: [displacement flux]
J = σE: [conduction current density]
B = μH: [magnetic flux density]

$$\begin{array}{l} \nabla \times H=E\left(j\varpi \left(ɛ\prime -jɛ\prime \prime \right)+\sigma \right)=\left(j\varpi ɛ\prime +\varpi ɛ\prime \prime +\sigma \right)E=j\varpi \left(ɛ\prime -jɛ\prime \prime -j\frac{\sigma }{\varpi }\right)E=j\varpi \left(ɛ\prime -j\left(ɛ\prime \prime +\frac{\sigma }{\varpi }\right)\right)E\\ \nabla \times H=j\varpi \left(ɛ\prime -j\left(ɛ\prime \prime +\frac{\sigma }{\varpi }\right)\right)E=j\varpi \left(ɛ\prime -j\frac{\varpi ɛ\prime \prime }{\varpi }+\frac{\sigma }{\varpi }\right)E=j\varpi \left(ɛ\prime -j\frac{\varpi ɛ\prime \prime +\sigma }{\varpi }\right)E\\ \nabla \times H=j\varpi \left(ɛ\prime -j\frac{\varpi ɛ\prime \prime +\sigma }{\varpi }\right)E=j\varpi ɛ\prime \left(1-j\frac{\varpi ɛ\prime \prime +\sigma }{ɛ\prime \varpi }\right)E=j\varpi ɛ\prime \left(1-j\;\text{tan}\;\delta \right)E \end{array}$$

where:

$$\text{tan}\;\delta \;\Rightarrow \frac{\varpi ɛ\prime \prime +\sigma }{\varpi }$$

Taking curl of both sides

$$\begin{array}{l} \nabla \times \left(\nabla \times E\right)=\nabla \times \left(-j\varpi \;\mu H\right)=-j\varpi \;\mu \left(\nabla \times H\right)=-j\varpi \;\mu \left(j\varpi ɛ\prime \left(1-j\frac{\varpi ɛ\prime \prime +\sigma }{ɛ\prime \varpi }\right)\right)E\\ \nabla \times \left(\nabla \times E\right)=\left(\mu \varpi^{2}ɛ\prime \left(1-j\frac{\varpi ɛ\prime \prime +\sigma }{ɛ\prime \varpi }\right)\right)E\\ \nabla \times \nabla \times E=\nabla \left(\nabla •E\right)-\nabla^{2}E \end{array}$$

Noting in a source free region ∇ • *E* = 0

$$\begin{array}{l} \nabla \times \nabla \times E=-\nabla^{2}E={\mu \varpi }^{2}ɛ\prime \left(1-j\frac{\varpi ɛ\prime \prime +\sigma }{ɛ\prime \varpi }\right)E=\left({\mu \varpi }^{2}ɛ\prime \left(1-j\;\text{tan}\;\delta \right)\right)E\\ \nabla^{2}E+{\mu \varpi }^{2}ɛ\prime \left(1-j\frac{\varpi ɛ\prime \prime +\sigma }{ɛ\prime \varpi }\right)E=0 \end{array}$$

In defining the wave-number k^2^, solution to the Helmholz wave equation, as the coefficient to “E”:

$$k^{2}={\mu \varpi }^{2}ɛ\prime \left(1-j\frac{\varpi ɛ\prime \prime +\sigma }{ɛ\prime \varpi }\right)$$

which then provides the propagation coefficient γ as:

$$\gamma =\mathrm{jk}=\alpha +j\beta =j\sqrt{{\mu \varpi }^{2}ɛ\prime \left(1-j\left(\frac{\varpi ɛ\prime \prime +\sigma }{\varpi ɛ\prime }\right)\right)}=j\varpi \sqrt{\mu ɛ\prime }\sqrt{\left(1-j\left(\frac{\varpi ɛ\prime \prime +\sigma }{\varpi ɛ\prime }\right)\right)}=j\varpi \sqrt{\mu ɛ\prime }\sqrt{\left(1-j\;\text{tan}\;\delta \right)}$$

which is used in the solution to the Helmholz wave equation, propagating in the plus z direction with magnitude E+, as:

$$E_{x}\;(z)=E^{+}e^{-\mathrm{jkz}}=E^{+}e^{-\gamma z}=E^{+}e^{-(\alpha +j\beta )z}=E^{+}e^{-\alpha z}e^{-j\beta z}$$

which can also be equivalently represented in the time domain as:

$$E_{x}\;(t,z)=E^{+}e^{-\alpha z}\text{cos}(\varpi \;t-\beta z)$$

from which we can see the phase of the wave is equal to:

θ=βz

In non-magnetic medium:

(2) $$\gamma =\alpha +j\beta =j\sqrt{\varpi^{2}\mu_{0}ɛ\prime (1-j\;\text{tan}\;\delta )}$$

where:

ɛ′ = real, dielectric constant, term of the complex permittivity (F/m).
ɛ″ = imaginary, loss, term of the complex permittivity (F/m).
γ = propagation coefficient (1/m).
α = attenuation factor of the propagation coefficient (nepers/m).
*j* = unit imaginary number √−1.
β = phase delay factor of the propagation coefficient (rads/m).
σ = conductivity factor of the propagation coefficient (S/m).
μ = material permeability (H/m).
*ϖ* = omega (rads/s).

Re-arranging Equation (2):

(3) $$\gamma =\alpha +j\beta =j\sqrt{\varpi^{2}\mu_{0}ɛ\prime (1-j\;\text{tan}\;\delta )}$$

(4) $$\gamma =\alpha +j\beta =j\varpi \sqrt{\mu_{0}ɛ\prime }{\left(1-j\;\text{tan}\;\delta )\right)}^{\frac{1}{2}}$$

Noting for soil where the abs(e″/e′) < 1, Equation (4) can be expanded via a power series approximation to:

$$\alpha +j\beta =j\varpi \sqrt{\mu_{0}ɛ\prime }{\left(1-j\;\text{tan}\;\delta )\right)}^{\frac{1}{2}}=j\varpi \sqrt{\mu_{0}ɛ\prime }\left(\sum_{n=0}^{\infty }\left(\begin{array}{c} \frac{1}{2}\\ n \end{array}\right)x^{n}\right)$$

$$\alpha +j\beta =j\varpi \sqrt{\mu_{0}ɛ\prime }\left(\begin{array}{l} 1+\frac{1}{2}\left(-j\;\text{tan}\;\delta \right)-\frac{1}{8}{\left(-j\;\text{tan}\;\delta \right)}^{2}+\frac{1}{16}{\left(-j\;\text{tan}\;\delta \right)}^{3}-\frac{5}{128}{\left(-j\;\text{tan}\;\delta \right)}^{4}+\\ \frac{7}{256}{\left(-j\;\text{tan}\;\delta \right)}^{5}-\frac{21}{1024}{\left(-j\;\text{tan}\;\delta \right)}^{6}+... \end{array}\right)$$

$$\alpha +j\beta =j\varpi \sqrt{\mu_{0}ɛ\prime }(1+\frac{1}{8}{\left(\text{tan}\;\delta \right)}^{2}-\frac{5}{128}{\left(\text{tan}\;\delta \right)}^{4}+\frac{21}{1024}{\left(\text{tan}\;\delta \right)}^{6}+j\left(\frac{1}{16}{\left(\text{tan}\;\delta \right)}^{3}-\frac{1}{2}\left(\text{tan}\;\delta \right)-\frac{7}{256}{\left(\text{tan}\;\delta \right)}^{5}\right))$$

(5) $$\alpha =\varpi \sqrt{\mu_{0}ɛ\prime }\left(\frac{1}{2}\left(\frac{\varpi ɛ\prime \prime +\sigma }{\varpi ɛ\prime }\right)-\frac{1}{16}{\left(\frac{\varpi ɛ\prime \prime +\sigma }{\varpi ɛ\prime }\right)}^{3}+\frac{7}{256}{\left(\frac{\varpi ɛ\prime \prime +\sigma }{\varpi ɛ\prime }\right)}^{5}\right)$$

(6) $$\beta =\varpi \sqrt{\mu_{0}ɛ\prime }\left(1+\frac{1}{8}{\left(\frac{\varpi ɛ\prime \prime +\sigma }{\varpi ɛ\prime }\right)}^{2}-\frac{5}{128}{\left(\frac{\varpi ɛ\prime \prime +\sigma }{\varpi ɛ\prime }\right)}^{4}+\frac{21}{1024}{\left(\frac{\varpi ɛ\prime \prime +\sigma }{\varpi ɛ\prime }\right)}^{6}\right)$$

Noting that for cotton, as well as typical soils, the largest dielectric loss occurs for heavy soils at saturation at the lower frequencies, which the simulation model provided by Peplinski and Dobson [20–22] suggests an e″/e′ ratio ≤ 0.25 typically and likely never more than e″/e′ ratio ≤ 0.5. Noting that with e″/e′ ≤ 0.5, the impact of the second and higher terms in Equation (5), for alpha, provides less than a 1% error. Similarly the third and higher order terms in Equation (6) can also be omitted, at the e″/e′ <= 0.5 ratio, with less than 0.5% error. Thus, a simplified solution for geo-science work provides Equations (7,8):

(7) $$\begin{array}{c} \alpha =\varpi \sqrt{\mu_{0}ɛ\prime }\left(\frac{1}{2}\left(\frac{ɛ\prime \prime }{ɛ\prime }\right)\right)=\sqrt{\mu_{0}ɛ\prime }\left(\frac{1}{2}\left(\frac{\varpi ɛ\prime \prime +\sigma }{ɛ\prime }\right)\right)==\sqrt{\mu_{0}ɛ\prime }\left(\frac{1}{2}\left(\text{tan}\;\delta \right)\right)\\ \alpha =\varpi \sqrt{\mu_{0}ɛ\prime }\left(\frac{1}{2}\left(\frac{ɛ\prime \prime }{ɛ\prime }\right)\right)=\varpi \sqrt{{ɛ}_{R}^{\prime }}\sqrt{\mu_{0}{ɛ}_{0}^{\prime }}\left(\frac{1}{2}\left(\frac{ɛ\prime \prime }{ɛ\prime }\right)\right)=\frac{\varpi \sqrt{{ɛ}_{R}\prime }}{c}\left(\frac{1}{2}\left(\frac{{ɛ}_{R}\prime \prime }{{ɛ}_{R}\prime }\right)\right) \end{array}$$

(8) $$\beta =\varpi \sqrt{\mu_{0}ɛ\prime }\left(1+\frac{1}{8}{\left(\frac{ɛ\prime \prime }{ɛ\prime }\right)}^{2}\right)$$

We also note that for e″/e′ ratios ≤ 0.25, typical for cotton and for soils on the dry end, the second term in Equation 8 can be omitted as the error is less than 1% and at e″/e′ ratios ≤ 0. 5 the error is less than 3% which allows for simplification of Equations (7,8), to the simplified form of Equations (9–11), for these special cases:

(9) $$\beta =\varpi \sqrt{\mu_{0}ɛ\prime }=\frac{\varpi }{c}\sqrt{{ɛ}_{r}\prime }$$

(10) $${ɛ}_{r}\prime =\frac{\beta^{2}c^{2}}{\varpi^{2}}=\frac{\beta^{2}}{\beta_{0}^{2}}$$

and:

$$\alpha =\varpi \sqrt{\mu_{0}ɛ\prime }\left(\frac{1}{2}\left(\frac{ɛ\prime \prime }{ɛ\prime }\right)\right)=\beta \left(\frac{1}{2}\left(\frac{ɛ\prime \prime }{ɛ\prime }\right)\right)=\beta \left(\frac{1}{2}\left(\frac{{ɛ}_{0}{ɛ}_{r}\prime \prime }{{ɛ}_{0}{ɛ}_{r}\prime }\right)\right)=\beta \left(\frac{1}{2}\left(\frac{{ɛ}_{r}\prime \prime }{{ɛ}_{r}\prime }\right)\right)$$

(11) $$ɛ\prime \prime =\frac{2\alpha {ɛ}_{r}\prime }{\beta }=\frac{2\alpha }{\beta }\frac{\beta^{2}}{\beta_{0}^{2}}=\frac{2\alpha \beta }{\beta_{0}^{2}}$$

**Figure 1.**
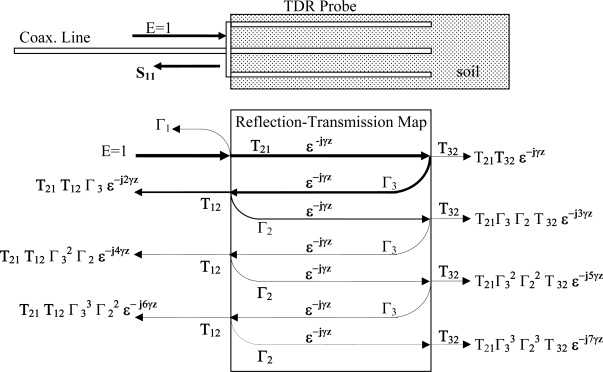
Detail of resultant waveform from combination of multiple reflections from both the leading edge, undesired, and probe end, desired measurement, in TDR/FDR probes due to impedance miss-match between coaxial cable impedance Zo to the soil-probe impedance Z1. Note: Hatched area indicates soil or other material under test.

Of note is that while for free-space wave propagation, Equations (1,2) are sufficient to provide a means to measure complex permittivity, however when measurements are taken with a TDR, or coaxial reflectance or through-transmission probe, the probe structure modifies how the plane wave propagates inside the coaxial waveguide. Thus, to obtain a measurement of absolute permittivity, rather than apparent permittivity, it is necessary to also characterize the waveguide’s effect on the plane-wave in order to extract the real permittivity from a measurement taken with the coaxial probe. We note that also of critical importance is the impedance miss-match between the coaxial cell and the interconnecting cable as the impedance miss-match setups up multiple reflections (Figure 1) which further confound the measurement.

To find the response when the medium is inside a coaxial cable, the formulation must transition from electric and magnetic fields to voltage, current and impedance. The impedance of a coaxial cable can be shown to be as in Equation (12) [14]:

(12) $$Z_{C}=\frac{\eta }{2\pi }\text{ln}(\frac{b}{a})$$

$$\eta =\sqrt{\frac{\mu_{0}}{ɛ}}$$

where:

*Z_C_* = impedance of the TDR probe with non-permeable medium and complex permittivity ɛ.
*η* = impedance of dielectric medium filling coaxial core between inner and outer conductors.
*b* = outer diameter of coaxial core
*a* = inner diameter of coaxial core.

Next we note that for a given geometry, *Z_C_* will not match *Z_0_* (impedance of the measurement system and inter-connecting cable). Due to this miss-match between *Z_C_* and *Z_0_*, a partial reflection of the incoming wave will take place at the front edge between the coaxial cable connector at the beginning of measurement zone. Thus, at the interface between the cable and coaxial soil-waveguide, there will be a reflection back towards the source. Further, the partially transmitted wave will then proceed to the end of the coaxial cell where it will reflect back towards the front edge where again the impedance miss-match will cause a partial reflection such that the wave again has to propagate through the cell a 2nd time and so on, which leads to multiple internal reflections, see Figure 1.

**Figure 2.**
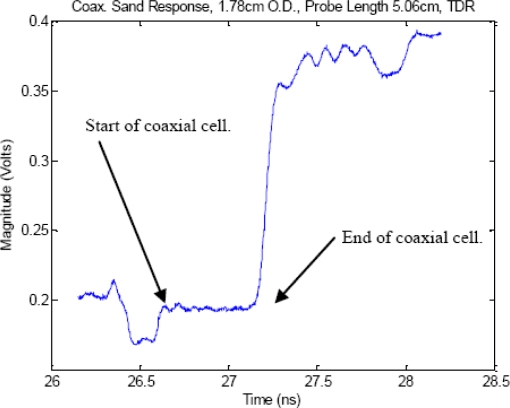
TDR experimental response for full coaxial cell loaded with dry sand with a near optimal impedance match between the coaxial cable and the TDR coaxial cell. The experimental response was due to the coaxial cell’s impedance being very close to 50 Ω. This matched condition was generated by using an outer diameter-to center rod diameter ratio that ensures the coaxial cell presents a near 50 Ω impedance to the system. This near 50 Ω matched condition preserves the sharp edges thereby improving accuracy of the measured TDR response.

**Figure 3.**
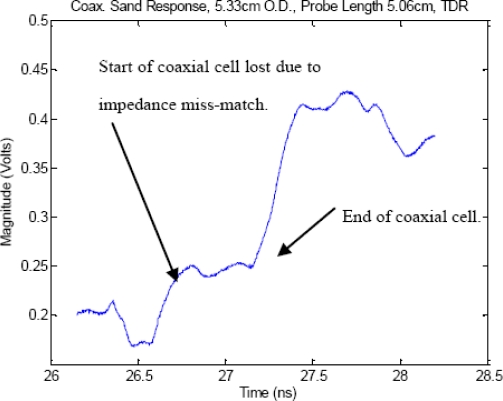
TDR experimental response for full coaxial cell loaded with dry sand with a large impedance miss-match between the coaxial cable and the TDR coaxial cell. The experimental response of the coaxial cell is constructed with the standard TDR diameter of 5 cm, which when loaded with dry sand, ɛ_r_ = 2.65, provides a coaxial impedance much higher than 50 Ω. This miss-matched impedance of the waveform is responsible for the rounding of the signal which in turns leads to measurement inaccuracies.

To examine this multiple impedance issue in closer detail, Figure 2 shows a comparison that the effect of impedance miss-match has on a TDR pulse’s reflected signal between when the cell is perfectly matched, and the large degradation in signal quality when the cell becomes miss-matched, which leads to large errors in estimation of the time delay which as Figure 3 shows, is caused by significant amounts of internally reflected energy, from the impedance miss-match, that sets up standing waves inside the coaxial cell, per Figure 1 reflection map. We note that T_32_ in the reflection map, is energy that is radiated out off the end of the probe which typically happens when the excitation signal’s wavelength is equal to ¼ of the TDR probe’s center conductor length (for the case where air is the medium surrounding the probe and at subsequently lower frequencies as the dielectric magnitude is increased).

For further examination of the reflection map, we note that the magnitude of the first reflection, Γ_1_ of Figure 1, that occurs at the interface between the coaxial cable and the coaxial cell, is due to an impedance miss-match, where the magnitude of the Γ_1_ reflection is provided in Equation (13) [17]:

(13) $$\Gamma_{1}=\frac{Z_{C}-Z_{0}}{Z_{C}+Z_{0}}$$

where:

Γ_1_ = reflection coefficient at transition from cable to TDR probe (Figure 1).
Z_0_ = impedance of the coaxial cable connecting soil probe to instrumentation.
Z_C_ = impedance of the coaxial soil probe, Equation (15(a)).

Similarly, the magnitude of the transmission coefficient, T_1_ can be found from continuity boundary conditions across the interface which leads to Equation (14) [17]:

(14) $$T_{1}=1+\Gamma_{1}$$

where T_1_ = transmission coefficient at transition from cable to TDR probe (Figure 1).

Similarly, the magnitude of the center probe end-point reflection, Γ_3_ of Figure 1, is provided in Equation (15):

(15) $$\Gamma_{3}=\frac{Z_{3}-Z_{C}}{Z_{C}+Z_{3}}$$

where:

Γ_3_ = reflection coefficient at transition from end of coaxial cell’s center probe to the soil beyond the coaxial cell, Figure 1.
Z_3_ = impedance due to radiation leakage from the end of the coaxial cell’s center probe.

We also note from Figure 1, which in addition to the first reflection Γ_1_ back towards the source, a cascading series of reflections takes place such that the sum of the multiple reflection combinations provides the measured frequency response reflection coefficient, S_11_ in Figure 1, that the coaxial cell measurement is actually providing, is detailed in Equation (16):

(16) $$\Gamma_{\mathrm{measured}}=\Gamma_{1}+T_{21}T_{12}\Gamma_{3}e^{-2\gamma z}+T_{21}T_{12}\Gamma_{2}\Gamma_{3}^{2}e^{-4\gamma z}+T_{21}T_{12}\Gamma_{2}^{2}\Gamma_{3}^{3}e^{-6\gamma z}+...$$

where:

γ = propagation constant for the medium, Equation (1).
z = propagation distance through the medium (m)
Γ_1_ = reflection coefficient off transition from cable to TDR probe, Figure 1.
Γ_2_ = reflection coefficient off transition from TDR probe to cable, Figure 1.
Γ_3_ = reflection coefficient off far tip of TDR probe, Figure 1.
T_12_ = transmission coefficient for the transition from coax. cable to coaxial-cell, Figure 1.
T_21_ = transmission coefficient for the transition from coaxial-cell back to coax. cable, Figure 1.

Noting a series expansion can be used to find the final form of the complete equation relating the measured reflection coefficient Γ_measured_ to the desired free space propagation constant γ required for determination of the true material permittivity is shown in Equation (17):

(17) $$\Gamma_{\mathrm{measured}}=\frac{\Gamma_{1}+\Gamma_{3}e^{-2\gamma z}}{1+\Gamma_{1}\Gamma_{3}e^{-2\gamma z}}$$

For comparison to Equation (17), the Clarkson [2,4] Equation is shown here as Equation (18):

(18) $$\Gamma_{\mathrm{measured}}=\frac{\Gamma_{1}+e^{-2\gamma z}}{1+\Gamma_{1}e^{-2\gamma z}}$$

Of particular note is the missing reflection term Γ_3_, which is the reflection coefficient off the end of the TDR or coaxial cell probe. Given the missing Γ_3_ term in the Clarkson Equation, and missing as well in the Kraft and Campbell formulation [7,8] and all the authors referencing those bodies of work, this entire body of work has a hidden built in assumption for perfect reflections, *i.e.*, Γ_3_ = 1, which is the only way for Equation (17) to reduce to the Clarkson [2] Equation, shown in Equation (18). However, of particular note here is that the hidden assumption of *Equation (18) is only valid for the case of perfect end reflection, i.e., if Γ_3_ = 1, which in turn is satisfied if and only-if the probe does not radiate*. Unfortunately, this assumption is not valid at frequencies approaching the quarter-wave length, as at the quarter-wave length this condition no longer satisfies a non-radiation condition, and Γ_3_ << 1 as T_32_, of Figure 1, as the probe is effectively acting as an antenna. Thus, of interest is if the Clarkson Equation (18), [2,7,8], must be replaced by Equation (17) along with some means to provide an estimate of Γ_3_ or T_32_ and if so, the magnitude of the errors involved. This paper will provide experimental evidence where it will be shown that modern 20 cm TDR probes begin to radiate above 0.9 GHz in air, which is well within the operating bandwidth of the typical TDR systems used in geophysical research, leading to the condition that Γ_3_ < 1, thus the underlying equation formulation is subject to hidden errors. It will also be shown that this radiation frequency decreases as the permittivity of the material surrounding the probe increases.

## 2. Experimental Section

Experimentally, of use in investigations of antenna radiation, is the “SWR” plot which is typically used for antenna design as it clearly shows when energy is being lost from the system and is being radiated out into space.

**Figure 4.**
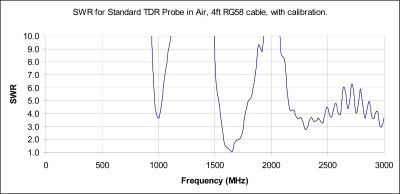
Frequency response of typical 20 cm TDR probe in air. We note that the “SWR” plot is typically used for antenna design as it clearly shows when energy is being lost from the system and is being radiated out into space.

Of concern for TDR measurements is when SWR is less than 10 as that occurs when more than 18% of the input power is not being returned back to the instrument for measurement, which if using the Clarkson or Kraft-Campbell equations, leads directly to the erroneous conclusion that the soil absorbed 18% of the energy, rather than the true answer that the probe is simply transmitting the energy out into space, or soil, away from the probe. Of note is that at an SWR equal 4, Γ_3_ = 0.6 which translates to 40% of the power is lost to radiation and is not being returned to the instrument for measurement. At the instrument, this false reduced return would then be erroneously interpreted as a very lossy material which in turn produces significant errors in the estimated delay and the calculation of the real portion of permittivity ɛ_r_’. As can be seen from the experimental test, shown in Figure 4, for a typical 20 cm TDR probe, at 1 GHz, Γ_3_ < 0.82 and at 1.65 GHz, also still well within the typical TDR instrument’s bandwidth, Γ_3_ < 0.05 and the TDR probe is acting as a very efficient radiator that is suitable for a telecommunications wireless link.

## 3. Results and Discussion

### TDR probe radiation violates basic the assumptions of the Campbell [8] and Clarkson [2] Equations:

The next question that naturally arises, given the experiment shown in Figure 4 that was performed in air; how does an increase in permittivity affect the radiation condition of the 20 cm TDR probe? To answer this, we note from micro-strip antenna design that the effective length, at which radiation occurs is affected by the neighboring substrate’s permittivity is shown in Equation (19) [16]:

(19) $$L_{\mathrm{effective}}\approx \frac{\lambda }{2}=\sqrt{{ɛ}_{r,\mathrm{effective}}}[L+H]$$

where:

λ = wavelength (m).
L_effective_ =effective length at which radiation condition occurs.
L = length of microstrip patch antenna (m).
H = height of dielectric substrate below microstrip patch (m).

Effectively, Equation (19) indicates that as the permittivity of the soil surrounding the TDR probe increases, so too does *L_effective_* which thereby increases the wavelength λ which in turn decreases the frequency that radiation occurs. This can be seen in detail from the relations between phase velocity, *v_p_*, the wavelength λ, and the frequency *f*, of a plane electromagnetic wave, provided here for convenience in Equations (20–22) [17]:

(20) $$v_{p}=\frac{c}{\sqrt{{ɛ}_{r}\;\mu_{r}}}$$

(21) $$\lambda =\frac{v_{p}}{f}$$

(22) $$f=\frac{c}{\lambda \sqrt{{ɛ}_{r}\;\mu_{r}}}$$

where:

c = speed of light (m/s)
ɛ_r_ = effective relative permittivity (F/m)
μ_r_ = effective relative permeability (H/m)
λ = wavelength (m)
f = frequency (Hz)
v_p_ = phase velocity (m/s)

**Figure 5.**
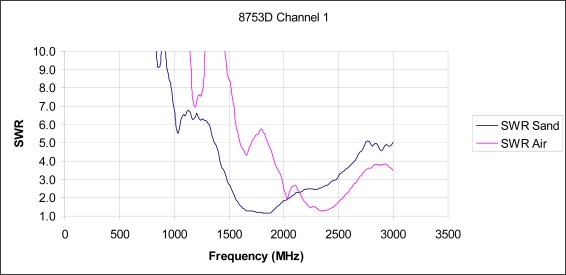
SWR comparison plot of quarter-wave insertion probes showing resonance locations for dry sand and air. Of particular note is the decrease in the resonant frequency that occurs as the permittivity of the material surrounding the TDR probe increases.

In summary, it can be seen from Equations (20–22), that as the soil surrounding the probe becomes wetter, with subsequently increasing permittivity ɛ_r_, the frequency at which returned power losses, due to antenna radiation from the TDR probe out into the soil, occurs, which happens at increasingly lower frequencies as soil moisture increases. To validate this hypothesis with respect to soil applications, Γ_3_ was measured both in air and then submerged in sand. As detailed in Figure 5, the resonant frequency does indeed shift to lower frequencies as the permittivity of the material surrounding the probe is increased.

**Figure 6.**
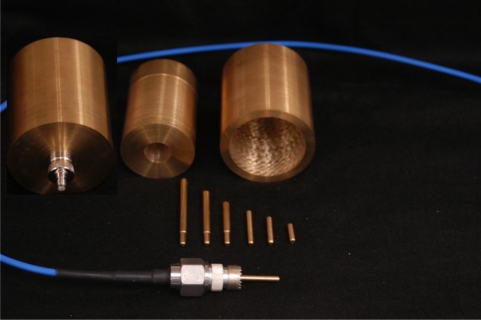
Machined brass coaxial cell based on commercial N to UHF (RF) adapter. System utilizes a brass machined insert, for the center probe, that allows for swapping in/out center probes, thereby providing a center-probe length change for the system, while maintaining the original calibration that removes the effects of the instrument, interfacing cable and the RF adapter. On the right is the large brass insert that provides a similar means to maintain the original system calibration while providing the means to alter the outer diameter of the coaxial cell, hence altering the impedance, of the coaxial cell. This system was designed, with the center insert installed, to provide a near perfect 50 Ω match for the coaxial cell when filled with dry sand ɛ_r_ = 2.85.

In the interest of obtaining guidance into the levels of expected accuracy that can be obtained by the proposed miss-match impedance correction protocol, in a non-radiating condition; experiments were conducted utilizing full coaxial cells machined out of brass (Figure 6). Brass was chosen as Kraft [7] has suggested that there may be some additional confounding effects due to the permeability of steel that is known to provide an added loss and hence an expected increase in the experimental errors. To remove the effects of radiation, the length of the probe was shortened to limit radiation to frequencies above 3.5 GHz. Finally, as Equation (3) indicates that a change in diameter will also cause a change in the obtained measured reflectance coefficient, the comparison of two otherwise identical coaxial cells with different outer-diameters to adjust the coaxial cell’s effective impedance was of interest.

Noting that for accurate utilization of a Network Analyzer, a major requirement is for the instrument connectors and cables to be calibrated out of the system. This calibration requirement however causes some difficulties in performing direct comparisons between the two like-coaxial cells, as ideally one would like to use the same calibration for both cells. In order to achieve this single calibration/dual use condition, a close fitting drop-in insert, designed to reduce the outer diameter, was machined to allow for direct comparison of two probe geometries without the need for changing connectors and the probe structure, thereby avoiding the need for a recalibration thereby enhancing the accuracy of the comparison.

The outer diameter of the large coaxial cell was chosen for similar dimensions to industry standard 20 cm TDR probes. The smaller diameter insert was chosen to give a 50 Ω impedance when the coaxial cell was filled with dry sand, with an estimated relative permittivity of ɛ_r_ = 2.85. Further in an effort to increase the confidence of the obtained results, multiple internal probes, the current carrying member of the inner diameter of the coaxial cell, were all machined at the same diameter and different lengths, for comparison of experimental results to the theoretically predicted values.

In the interest of restricting the internal reflections to only those of the model of Figure 1, care was taken in the construction of the coaxial cell to coaxial cable interface. The research eventually identified that female UHF to SMA coaxial adapters provide a near ideal interface as the female UHF adapter is already threaded with 5/8–25 threads that provide a means for obtaining a high-quality coupling to a machined and like-threaded coaxial cell while providing a commercial quality rf connection between the coaxial cell and the coaxial cable. Upon investigation of the proposed setup (Figure 6) the TDR results (Figure 2) show the coaxial cell provides a clean transition, for the matched configuration, from the coaxial cable to the coaxial cell with only a minor reflection occurring mainly inside the UHF to SMA transition which is easily removed from the measurements through a standard short-open-load one-port error correction calibration protocol on the network analyzer.

For the network analyzer calibration, referenced earlier, the research used an open-short-load protocol to move the reference plane to the location of the short. Some experimentation on calibration for this system, quickly lead to the realization that a choice had to be made between using either an in-house built shorting element that provides the short at the correct location, thereby establishing the reference plane correctly; or utilize a high-quality commercial short, designed specifically for calibrations, that would inadvertently put the calibration plane in the wrong location, thereby leading to a phase error that would have to be corrected via a model. Further as the calibration standards are based on very high quality N connectors, there is also an error introduced by the UHF connector itself. After running some preliminary experiments, the results suggested that for the highest accuracy work, a well designed in-house constructed short made from an identical N-UHF adapter, identical to the one used in interfacing to the coaxial cell, provided the best results.

### Missing factors in Clarkson and Campbell Equations

As a first comparison into the validity of the miss-match impedance correction technique, we note the work by Heimovarra [15], that suggests an accurate measurement can be made without an impedance miss-match at the ¼ wavelength frequency as the impedance at the ¼ wavelength frequency is 50 Ω, hence they suggest that at this one special frequency, the measurement is correct without correction. This observation by Heimovarra, [15], is also well accepted in the microwave engineering field and is commonly utilized in the technique of impedance matching using a ¼ wavelength line [17].

Noting that Equation (3) indicates that the permittivity of the material as well as the outer diameter of the coaxial cell affects the impedance, Equations (12,13) show both the permittivity and diameter subsequently affect the internal reflection coefficients, Γ_1_ Γ_3_, that dictate the measured reflection, repeating Equation (17) here for convenience:

(17) $$\Gamma_{\mathrm{measured}}=\frac{\Gamma_{1}+\Gamma_{3}e^{-2\gamma z}}{1+\Gamma_{1}\Gamma_{3}e^{-2\gamma z}}$$

For the case where the system is almost matched at the ¼ wavelength line length, the discontinuities between impedances are small yielding Γ_1_ Γ_3_ << 1. Thus, for small changes in impedances between the cable and the coaxial cell, Equation (17), at the ¼ wavelength frequency, can be approximated by Equation (23).

(23) $$\Gamma_{\mathrm{measured}}=\Gamma_{1}+\Gamma_{3}e^{-2j\gamma z}$$

Further noting that by definition that at the ¼ wavelength frequency, the following condition is true (assume low loss condition):

(24) $$\gamma z=(\alpha +j\beta )z=\frac{\pi }{2}$$

Thus at the ¼ wavelength frequency, for the non-radiating conditions *Γ_3_= 1*, Equation (17) reduces to:

(25) $$\Gamma_{\mathrm{measured}}=\Gamma_{1}+e^{-j\pi }=\frac{Z_{1}-Z_{0}}{Z_{1}+Z_{0}}+e^{-j\pi }$$

Noting that in the matched condition:

(26) $$Z_{C}=Z_{0}$$

then:

(27) $$\Gamma_{\mathrm{measured}}=-1∠\pi$$

Thus, when the phase of the reflected wave is delayed by π radians, the frequency of this occurrence corresponds to the ¼ wavelength matched condition. Further noting that at the matched ¼ wavelength condition:

(28) $$Z_{C}=Z_{0}=\frac{1}{2\pi }\text{ln}(\frac{b}{a})\sqrt{\frac{\mu_{0}}{ɛ}}$$

Rearranging Equation (28) provides Equation (29):

(29) $$b=a\;{\text{exp}}^{\left(2\pi ɛZ_{0}\sqrt{\frac{1}{\mu_{0}}}\right)}$$

Equation (29) is significant as it clearly indicates that when the coaxial cell is matched via a ¼ wavelength resonant impedance transformer, for a given ɛ permittivity, there is only one outer diameter that will provide a matched condition. Thus, for all other ɛ permittivity’s, and soil moisture contents, the coaxial cell will be miss-matched and will require correction via an inverse solution to Equation (18), for the non-radiating case or via Equation (17) for the radiating case which is required for the typical TDR installation especially when the soil is below 50% maximum allowable depletion.

For the purposes of relating this theory to experimentally derived measurements, some basic test cases utilizing low loss materials were investigated. For the low loss, Γ = 0, test cases and for true TEM lines at ¼ wavelength, which occurs at the λ/4 phase delay which indicates the resonant frequency with a matched condition, provides a direct measure of the propagation coefficient γ, as shown in Equation (30):

(30) $$\gamma z=\beta z=\frac{\pi }{2}=\beta \left(\frac{\lambda }{4}\right)=\frac{2\pi f}{v_{p}}\left(\frac{\lambda }{4}\right)=2\pi f\frac{\sqrt{\mu_{r}{ɛ}_{r}}}{c}\left(\frac{\lambda }{4}\right)$$

Rearranging (29) provides Equation (30), which provides the theoretical low loss resonant frequency for a given probe length equal to λ and relative permittivity ɛ_r_.

(30) $$f_{\mathrm{resonance}}=\frac{c}{\lambda \sqrt{\mu_{r}{ɛ}_{r}}}$$

Of particular note, is that Equation (30) is not dependent upon the coaxial cell’s geometry. Calculations utilizing Equation (17) to find the location of the ¼ wavelength resonant frequency for the air filled coaxial cell, shows the theory predicts the same location for the smaller inner-diameter coaxial cell as well as the larger-diameter coaxial cell. However, experimental results clearly show a difference, in the location of the ¼ wavelength resonant frequency, between the smaller diameter coaxial cell with respect to the larger diameter coaxial cell, when air or dry sand (low loss material) is placed inside the coaxial cell. It was also noted that the deviation in frequency between the two diameter cells, all other parameters remaining constant, is over 100 MHz. Thus, there must be some other effect, that is not accounted for in the theory, as presented thus far in this paper. We note herein, that the typical TDR approach to calibrating to air and water for a correction factor, has effectively hidden this error and while over the full range of soil permittivity’s ranging from e′ = 2.9 (dry sand) to e′ = 35 (saturated soil), the interpolation errors can in some cases be ignored when the calibration is used. However, for low loss media and for applications where the primary interest is on the dry end and for high accuracy applications, such as detection of water transport via roots, they cannot be ignored and a solution must be found that resolves these discrepancies. It should also be noted that this large a difference in the measured resonant frequency carries over to all frequencies and affects the accuracy of the obtained permittivity even after correction by the Clarkson [2] or Campbell [8] Equations, or as noted earlier, use of Equation (17) when dealing with a radiation condition. As an example, at 350 MHz, the measured response with the large diameter coaxial cell was a delay of 42 degrees, however with the small coaxial cell, the delay was reduced to only 36 degrees, a difference of 16%. Thus, for high accuracy absolute permittivity sensing, there is a critical need for resolving this issue especially for low loss materials and will be the subject of future work with the hypothesis proposed here that the unaccounted for error may be due to fringe capacitance off the end of the probe tip.

## 4. Conclusions

The results of this research, reported herein, indicate that due to radiation effects of the standard TDR probe, these probes are not suitable for measurement of permittivity beyond 900 MHz, which suggests the need to move to a shorter probe design when used for measurement of dry sandy soils where it is known that the frequency content of the reflected pulse will contain frequencies well in excess of 1,000 MHz.

The analysis of the Clarkson [2], and equivalently the Kraft [7] and Campbell [8] Equations, when used to correct for multiple reflections, were shown that these equations have an inherent assumption that there is a perfect reflection off the end of the TDR probe tip and thus the radiation effects, as discussed in the previous paragraph and detailed in this report all make the erroneous assumption that Γ_3_ = 1, Figure 1. Thus, the assumption in the Clarkson, Kraft and Campbell Equations are not valid and lead to increasingly larger errors as the soil becomes drier and the effective frequency increases past the resonant frequency of the TDR probe. We also note that for drier soils, where the frequency content exceeds 1,000 MHz in a TDR measurement, there is no way to perform an absolute measurement of permittivity, without a redesign of the TDR probe, as there is no means to separate out losses due to high conductance soils, such as occurs in high salinity regions or with fertigation techniques where the irrigation water is actively transporting salts to the root zone.

It is also note-worthy that as the level of permittivity in the soil increases, the frequency at which a radiation condition occurs becomes increasingly lower. Thus, in wet soils, ɛ_r_ ≥ 25, the evidence presented herein, suggests the effective TDR frequency, above which the Clarkson, Kraft and Campbell Equations [2,7,8] are no longer valid, is likely to occur at as low a frequency as 150 MHz. Thus, any method, frequency domain or time domain, that utilizes frequencies that exceed 150 MHz for very wet soils, will likely be significantly altered by the radiation condition which suggests the need for a radical redesign and/or shortening of the TDR probe, when the application demands high accuracy measurements utilizing frequency domain techniques for the measurement of absolute permittivity. As it is noted that the microbial actions resulting in increased N_2_O and H_2_S emissions from the soil occur in anaerobic conditions, this is a critical portion of the soil moisture regime for research into greenhouse gas emissions research and is an area of concern.

The research highlights that while the Clarkson [2], Kraft [7] and Campbell [8] equations do provide a correction that can be used to correct for multiple reflections, due to impedance miss-match, these equation are only valid for non-radiating frequencies (Figure 5). It is also noted that there is also likely a need for further research to confirm that a length correction to account for fringe capacitance error may also be required for high accuracy work in drier soils and other media of low permittivity and provides the impetus for future work.
